# Real‐World Treatment Patterns, Burden of Illness, and Survival of Autologous Stem Cell Transplant‐Ineligible Patients With Mantle Cell Lymphoma in Ontario, Canada

**DOI:** 10.1002/jha2.70384

**Published:** 2026-10-15

**Authors:** Sydney George, Soo Jin Seung, Sandra Thibault, Anisia Wong, Mackenzie A. Hamilton, Anca Prica

**Affiliations:** ^1^ Scientific Affairs BioPharmaceuticals, AstraZeneca Mississauga Canada; ^2^ Health Outcomes and PharmacoEconomics (HOPE) Research Centre Sunnybrook Research Institute North York Canada; ^3^ University Health Network Toronto Canada

**Keywords:** burden of illness, clinical outcomes, mantle cell lymphoma, real‐world evidence, transplant‐ineligible, treatment patterns

## Abstract

**Introduction:**

Since the approval of ibrutinib in 2016 in Canada for relapsed or refractory (R/R) mantle cell lymphoma (MCL), there has been limited evidence characterizing treatment patterns, burden of illness, and survival in Canadian patients ineligible for autologous stem cell transplant (ASCT). This is the first study in Ontario reporting real‐world treatment patterns and clinical outcomes of elderly patients with MCL who are transplant‐ineligible in 1L and R/R settings.

**Methods:**

The aim of this study was to characterize the real‐world use and outcomes of patients with MCL who were ≥ 65 years old and transplant‐ineligible in first‐line (1L) and R/R settings between 2013 and 2021 using administrative databases in Ontario, Canada. Incidence of MCL, treatment patterns, and overall survival (OS) were assessed.

**Results:**

The study cohort consisted of 754 patients (follow‐up: 36.1 months, median age: 76 [IQR: 71–82], 71.1% male). Most patients received bendamustine and rituximab in 1L (76.7%) and ibrutinib in second‐line (2L; 73.2%). Mortality rates following 1L and 2L therapy were 39.3% and 68.5%, respectively. Duration of therapy in 1L and 2L treatment was 4.70 and 2.14 months, respectively. Median OS decreased with subsequent lines of therapy (1L: 3.51 years; 2L: 0.60 years; third‐line: 0.43 years).

**Conclusion:**

This study characterized the treatment patterns, rwOS, and TTNT‐D of MCL patients in Ontario, Canada. Poor real‐world clinical outcomes support the need for additional 1L and R/R therapies for MCL management for an ASCT‐ineligible population.

**Trial Registration:**

The authors have confirmed clinical trial registration is not needed for this submission.

## Graphical abstract

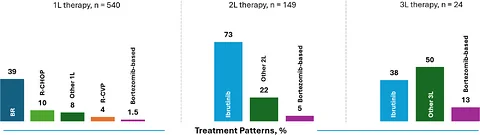

## 1 Introduction

Mantle cell lymphoma (MCL) is a rare and aggressive form of non‐Hodgkin lymphoma (NHL) [1]. In Canada, an estimated 500–600 people are newly diagnosed with MCL each year, accounting for 3%–10% of new NHL cases [1, 2, 3, 4]. MCL typically begins with slow growth (indolent disease) within lymph nodes, and eventually progresses to rapid growth and spread throughout the body [5]. Diagnosis often occurs at an advanced stage, which may involve the gastrointestinal tract, spleen, and bone marrow [5, 6, 7]. Most patients are diagnosed between 60 and 70 years of age [8].

In Ontario, standard of care first‐line (1L) treatment for MCL includes intensive chemo immunotherapies (CITs) with potential autologous stem cell transplant (ASCT) and rituximab maintenance treatment. This approach is usually reserved for younger, fit patients. Patients over 65 are typically ineligible for intensive CITs due to the risk of toxicity and morbidity associated with these treatments, particularly ASCT [5, 9]. 1L treatment in this older cohort usually involves less intensive induction chemoimmunotherapy regimens, such as rituximab, cyclophosphamide, doxorubicin, vincristine, and prednisone (R‐CHOP); bendamustine and rituximab (BR); and vincristine, prednisone, and rituximab (R‐CVP); followed by maintenance with rituximab [10, 11]. The standard of care for patients with treatment‐naïve MCL requiring less aggressive therapy includes BR, established by the pivotal STiL trial that demonstrated longer progression‐free survival (PFS) and fewer toxic effects with BR compared to R‐CHOP [12, 13].

Despite available treatments, over half the patients with MCL develop relapsed or refractory (R/R) disease [1, 14]. Treatment options for R/R MCL include Bruton tyrosine kinase inhibitors (BTKis), CITs, CAR T‐cell regimens, and rarely, allogenic stem cell transplant, with access varying across provinces due to differences in funding criteria and approval statuses [5]. BTKis as 1L treatment have been investigated in older patients with MCL due to efficacy observed in R/R settings, reduced efficacy of less intensive CITs, and transplant‐ineligibility among older patients. The Phase III SHINE trial investigated a first‐generation BTKi, ibrutinib in combination with BR and rituximab maintenance, compared to placebo plus BR and rituximab maintenance in older patients with previously untreated MCL, demonstrating extended PFS (median PFS: 81 vs. 53 months) [15].

Similarly, the Phase III ECHO trial investigated a second‐generation BTKi, acalabrutinib, in combination with BR, compared to placebo plus BR in older patients with treatment‐naïve MCL. The interim analysis from ECHO demonstrated extended PFS with the acalabrutinib regimen (median PFS: 66 vs. 50 months) [16]. In addition, patients receiving the BTKi regimen in ECHO had lower discontinuation rates due to adverse events, serious adverse events, and no deaths due to cardiac disorders compared to SHINE [15, 16].

Since the approval of ibrutinib for patients with R/R MCL in 2016 in Canada, there has been limited real‐world evidence characterizing evolving treatment patterns and burden of illness in Canadian patients with treatment‐naïve and treatment‐exposed MCL, especially among those who are ASCT‐ineligible [17]. Therefore, the objective of this study was to characterize the real‐world treatment patterns and clinical outcomes of patients with newly diagnosed MCL who were ≥ 65 years old and transplant‐ineligible in 1L and R/R settings in Ontario, Canada.

## 2 Materials and Methods

### 2.1 Study Design

A retrospective, observational, population‐based study was conducted using linked health administrative data from the Institute for Clinical Evaluative Sciences (ICES) in Ontario, Canada. Patients who were ≥ 65 years of age and diagnosed with MCL (International Statistical Classification of Diseases [ICD]‐O‐3 morphology code for MCL [9673/3]) between January 1, 2013 and December 31, 2021 were included. Patients were followed up until December 31, 2022, to allow for a minimum follow‐up of 1 year (Figure S1). The index date was defined as the date of MCL diagnosis. Look‐back windows of 2 years prior to index were used to assess baseline characteristics, and 5 years prior to index to identify previous cancer diagnoses.

### 2.2 Patient Population

Patients included had a diagnosis of MCL as documented in the Ontario Cancer Registry (OCR), and were ≥ 65 years old at diagnosis. This age group was generally considered ASCT‐ineligible and received less intensive chemotherapy due to higher toxicity risk and comorbidities reflecting Ontario clinical practice.

Patients were excluded if they met any of the following criteria: < 65 years old at index, received ASCT (indicated by cross‐referencing with ICD‐10 diagnosis code Z52.011) at any time during follow‐up, invalid or incomplete records, or had not interacted with the health care system in the past 10 years or in the first year of follow‐up. Patients were also excluded if they had an invalid ICES Key Number or were ineligible for the Ontario Health Insurance Plan (OHIP).

### 2.3 Data Sources

Ontario's healthcare system provides publicly funded physician services, laboratory testing, and hospital care for all residents with a provincial health card (population ∼14.7 million). All healthcare interactions are housed and linked at ICES. Detailed descriptions are provided in Table S1.

The study was approved by the Sunnybrook Health Sciences Centre Research Ethics Board (REB #6410) and performed in compliance with Good Clinical Practice (GCP) and Good Pharmacoepidemiology Practice (GPP). No patient recruitment or consent was required.

### 2.4 Study Objectives and Outcomes

The primary objective was to describe treatment patterns by line of therapy and categorical type of therapy received per line in patients who were ≥ 65 years old with ASCT‐ineligible MCL. Secondary objectives were to describe the annual incidence of MCL, clinical and demographic characteristics, overall survival (OS), and time to next treatment or death (TTNT‐D).

1L treatment initiation was defined as the date of the first claim for any MCL‐specific antineoplastic agent following diagnosis, and included all antineoplastic agents received within the first 28 days of 1L therapy. Cases where 1L treatment had a < 90‐day gap and was re‐initiated as the same treatment were considered continued 1L treatment. Second‐line (2L) treatment initiation was defined as initiation of a subsequent treatment different from 1L, or initiation of the same treatment after at least a 90‐day gap. Any antineoplastic agent received within the first 28 days of the subsequent treatment line was considered part of the 2L regimen. Maintenance rituximab therapy was defined as receipt of single‐agent rituximab following 1L induction therapy, administered once every 84 days, up to 8 times.

The time to initial MCL treatment was defined as the time from the index date to the start date of the first treatment of systemic therapy for MCL. Duration of treatment was defined as the time from the first date of treatment to last date of treatment per therapy type by line of therapy.

### 2.5 Statistical Analysis

The annual incidence of patients with transplant‐ineligible MCL aged 65 and older was estimated as the number of documented diagnoses each year, divided by the general population aged 65 and older on July 1 of that same year. OS and TTNT‐D were estimated using Kaplan–Meier methods and reported using median and 95% confidence intervals (CIs). OS was defined as time from treatment initiation to death among patients who received systemic therapy. Death that occurred within 30 days of a positive SARS‐CoV‐2 laboratory test was considered death due to COVID‐19. TTNT‐D was defined as the time from treatment initiation to first date of subsequent treatment, death, or censoring. Censoring was defined as the last day of available follow‐up, date of death, or end of study period (December 31, 2022), whichever occurred first. Categorical treatments received in 1L and 2L settings were summarized using counts and proportions.

Data analysis occurred between October 2024 and May 2025 by ICES Data & Analytics Services (ICES DAS) Private Sector, using SAS Enterprise Guide 8.3 (SAS Institute Inc., Cary, NC, USA). Number ranges were used to avoid back calculation as per ICES DAS policies. If the number of patients receiving each type of treatment was five or less, certain treatment groups were combined to avoid small cells.

## 3 Results

### 3.1 Clinical and Demographic Characteristics

A total of 754 patients were included (Table 1, Figure S2). The mean duration of follow‐up was 36.1 months (standard deviation [SD]: 31.1). The median age at index date was 76 (interquartile range [IQR]: 71–82), and most patients were ≥ 75 years old (56.4%). The majority were male (71.1%), had no previous cancer diagnosis in the last 5 years (88.9%), and had Stage IV disease (22.4%) at diagnosis.

**TABLE 1:** Baseline demographic and clinical characteristics of patients diagnosed with MCL between January 1, 2013 and December 31, 2021.

Patient demographics and clinical characteristic	Statistic (*N* = 754)
Total duration of follow‐up (months), mean (SD)	36.1 (31.1)
Age at index date, median (IQR)	76 (71–82)
65 – 74, *n* (%)	329 (43.6%)
75+, *n* (%)	425 (56.4%)
Gender distribution (*N*, %)	
Male	536 (71.1%)
Female	218 (28.9%)
Residence (*N*, %)	
Rural	92 (12.2%)
Urban	659 (87.4%)
Missing	3 (0.4%)
Income quintile (*N*, %)	
1 (lowest)	130 (17.2%)
2	152 (20.2%)
3	159 (21.1%)
4	140 (18.6%)
5 (highest)	170 (22.5%)
Missing	3 (0.4%)
Collaborative staging at diagnosis (*N*, %)	
I	21 (2.8%)
II	13 (1.7%)
III	68 (9.0%)
IV	169 (22.4%)
V	0 (0.0%)
Missing	483 (64.1%)
Type of diagnosing centre (*N*, %)	
Community	441 (58.5%)
Academic	291 (38.6%)
Missing	22 (2.9%)
Previous cancer diagnosis (*N*, %)	
Yes (last 5 years)	84 (11.1%)
No (last 5 years)	670 (88.9%)
Charlson comorbidity score (*N*, %)	
0	66 (8.8%)
1	43 (5.7%)
2	31 (4.1%)
3+	41 (5.4%)
Not applicable	573 (76.0%)

Abbreviations: ICES = Institute for Clinical Evaluative Sciences; IQR = interquartile range; LHIN = Local Health Integration Network; MCL = mantle cell lymphoma; SD = standard deviation.

### 3.2 Incidence of MCL

The number of new transplant‐ineligible MCL diagnoses per year was consistent and ranged from 76 diagnoses in 2016 to 91 diagnoses in 2017 (Table 2). Estimated size of the population aged ≥ 65 years old increased every year from 2013 to 2021. Thus, the crude incidence rate of MCL was estimated to range from 0.0425 per 1000 in 2013 to 0.0332 per 1000 in 2021.

**TABLE 2:** Crude incidence of MCL among Ontarians ≥ 65 years old between 2013 and 2021.

Year	MCL diagnoses (*N*)	65+ Population estimate^a^ (*N*)	Crude Incidence per 1000 (95% CI)
2013	87	2,048,679	0.0425 (0.0344–0.0524)
2014	77	2,120,925	0.0363 (0.0290–0.0454)
2015	80	2,188,168	0.0366 (0.0294–0.0455)
2016	76	2,261,224	0.0336 (0.0268–0.0421)
2017	91	2,341,689	0.0389 (0.0316–0.0477)
2018	85	2,423,015	0.0351 (0.0284–0.0434)
2019	82	2,512,374	0.0326 (0.0263–0.0405)
2020	87	2,601,101	0.0334 (0.0271–0.0413)
2021	89	2,684,143	0.0332 (0.0269–0.0408)

Abbreviations: CI = confidence interval; MCL = mantle cell lymphoma.

^a^ Population aged 65+ residing in Ontario, eligible for OHIP, without missing birth date or sex, as of July 1st of the year of interest.

### 3.3 Treatment Patterns by Line of Therapy

A total of 540 patients (71.6%) were treated with systemic therapy, and 214 patients (28.4%) did not receive systemic therapy during follow‐up (Table 3). Some of the cohort (*n* = 168, 22.3%) received local radiotherapy, and 139 patients received both radiotherapy and systemic therapy.

**TABLE 3:** Treatment patterns in patients ≥ 65 years old diagnosed with MCL between 2013 and 2021.

Treatment use, *n* (%)	Overall (*N* = 754)
Lines of therapy received	
No systemic therapy	214 (28.4%)
Treated with systemic therapy	540 (71.6%)
1 only	391 (72.4%)
2 only	125 (23.2%)
3+	24 (4.4%)
Radiotherapy use	
Yes	168 (22.3%)
No	586 (77.7%)
Distribution of 1L therapy received	*n* = 540
BR	414 (76.7%)
R‐CHOP	54 (10%)
R‐CHOP/R‐DHAP	8 (1.5%)
R‐CVP	23 (4.3%)
Other^a^	41 (7.6%)
Distribution of 2L therapy received	*n* = 149
Ibrutinib	109 (73.2%)
Bortezomib‐based	8 (5.4%)
Other^b^	32 (21.5%)
Distribution of 3L therapy received	*n* = 24
Ibrutinib	9 (37.5%)
Bortezomib‐based	1–5^d^
Other^c^	10–14^d^
Receipt of maintenance rituximab therapy	
After 1L	277 (51.3%)
After 2L	0 (0%)
Death following treatment, prior to subsequent treatment	
Following 1L	212 (39.3%)
Death due to COVID‐19	7 (3.3%)
Following 2L	102 (68.5%)
Death due to COVID‐19	6 (5.9%)

Abbreviations: 1L = first line; 2L = second line; 3L = third line; BAC‐R = rituximab, bendamustine, and cytarabine; BR = bendamustine and rituximab; CHOP = cyclophosphamide, hydroxydaunorubicin, oncovin, and prednisone; ICES = Institute for Clinical Evaluative Sciences; MCL = mantle cell lymphoma; R‐CHOP = rituximab, cyclophosphamide, hydroxydaunorubicin, oncovin, and prednisone; R‐CVP = rituximab, cyclophosphamide, vincristine sulfate, and prednisone; R‐DHAP = rituximab, dexamethasone, cytarabine, and cisplatin.

^a^Other 1L: *bendamustine, *CHOP, chlorambucil + rituximab, R‐BAC, ibrutinib (only when it's the first treatment given), and rituximab (maintenance)/*rituximab (only when the regimen appears as a monotherapy, and there is no subsequent treatment to be combined with).

^b^Other 2L: R‐CVP, R‐CHOP, *CHOP, chlorambucil + rituximab, BR.

^c^Other 3L: BR, *CHOP, *bendamustine, CHOP+R.

^d^Small cell suppression in accordance with ICES privacy policy.

The most common systemic therapy received in 1L was BR (*n* = 414, 76.7%), followed by R‐CHOP (*n* = 54, 10%) and R‐CVP (*n* = 23, 4.3%) (Table 3). Few patients (*n* = 49, 9.1%) received other therapies in 1L (Table 3). A total of 277 (51.3%) patients received rituximab maintenance therapy after 1L. Following 1L, 212 (39.3%) patients died prior to 2L treatment, with 7 (3.3%) patients dying due to COVID‐19.

A total of 149 patients received 2L treatment (Figure 1). The most common 2L therapy was ibrutinib (*n* = 109, 73.2%), followed by bortezomib‐based regimens (*n* = 8, 5.4%) (Table 3). Some patients (*n* = 32, 21.5%) received other treatments in 2L (Table 3). No patients received rituximab maintenance therapy after 2L. Following 2L, 102 (68.5%) patients died prior to 3L treatment, with 6 (5.9%) patients dying due to COVID‐19.

**FIGURE 1:**
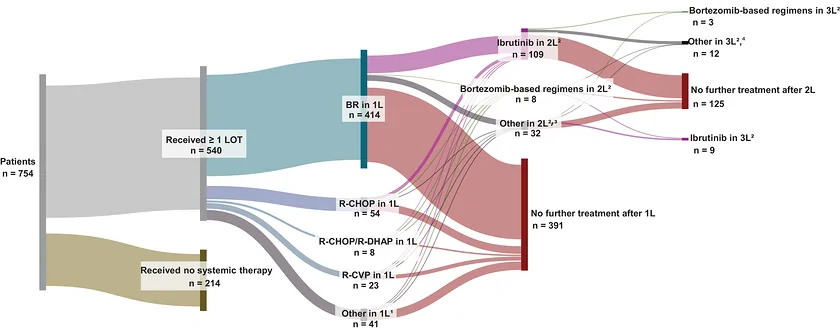
Treatment sequencing of patients ≥ 65 years old diagnosed with MCL between 2013 and 2021. Note that for data with small cell suppression, the midpoint value of the frequency range was used as a data input. ^1^Other 1L: (*bendamustine, *CHOP, CHLO + rituximab, R‐BAC, ibrutinib [only when it is the first treatment given], and rituximab [maintenance]/*rituximab [only when the regimen appears as a monotherapy, and there is no subsequent treatment to be combined with]). ^2^Other 2L: (R‐CVP, R‐CHOP, *CHOP, BR). ^3^Other 2L: (R‐CVP, R‐CHOP, *CHOP, CHLO + rituximab, BR). ^4^Other 3L: BR, *CHOP, *bendamustine, CHOP+R. 1L = first line; 2L = second line; 3L = third line; BAC‐R = rituximab, bendamustine, and cytarabine; BR = bendamustine and rituximab; CHLO = chlorambucil; CHOP = cyclophosphamide, hydroxydaunorubicin, oncovin, and prednisone; R‐CHOP = rituximab, cyclophosphamide, hydroxydaunorubicin, oncovin, and prednisone; R‐CVP = rituximab, cyclophosphamide, vincristine sulfate, and prednisone; R‐DHAP = rituximab, dexamethasone, cytarabine, and cisplatin.

Finally, 24 patients received third‐line (3L) treatment (Figure 1). In 3L, the most common therapy was ibrutinib (*n* = 9, 37.5%), followed by bortezomib‐based regimens (*n* = 1–5) (Table 3). Between 10 and 12 patients received other therapies in 3L (Table 3).

The median time from diagnosis to treatment initiation was 1.64 months (IQR: 0.99–3.88) for 1L and 21.6 months (IQR: 11.1–38.9) from diagnosis to 2L (Table S2). The median 1L treatment duration was 12.8 months (IQR: 4.64–26.4) with rituximab maintenance therapy and 4.70 months (IQR: 3.68–5.33) without rituximab maintenance therapy. The median 2L treatment duration was 2.14 months (IQR: 0.79–5.95), where median duration of ibrutinib in 2L was 2.07 months (IQR: 0.69–8.22).

### 3.4 Time to Next Treatment or Death

The median overall TTNT‐D was 2.62 years (95% CI: 2.24, 3.01) (Table 4). Patients receiving BR in 1L had the longest TTNT‐D (median: 2.85 years, 95% CI: 2.54, 3.62), followed by R‐CHOP (median: 1.74 years, 95% CI: 1.09, 3.21), and other 1L therapeutic regimens (median: 0.99 years, 95% CI: 0.61, 2.21) (Table 4; Figure S3).

**TABLE 4:** Time to next treatment or death from 1L to 2L treatment, by 1L treatment received.

	Median (95% CI), years
1L treatment	2.62 (2.24, 3.01)
BR	2.85 (2.54, 3.62)
R‐CHOP	1.74 (1.09, 3.21)
Other^a^	0.99 (0.61, 2.21)
R‐CHOP/R‐DHAP	(S)
R‐CVP	0.90 (0.52, 2.68)
Other	1.04 (0.41, 2.62)

*Note*: (S) suppressed as a result of small cell.

Abbreviations: 1L = first line; 2L = second line; BAC‐R = rituximab, bendamustine, and cytarabine; BR = bendamustine and rituximab;

CHLO = chlorambucil; CHOP = cyclophosphamide, hydroxydaunorubicin, oncovin, and prednisone; R‐CHOP = rituximab, cyclophosphamide, hydroxydaunorubicin, oncovin, and prednisone; R‐CVP = rituximab, cyclophosphamide, vincristine sulfate, and prednisone; R‐DHAP = rituximab, dexamethasone, cytarabine, and cisplatin.

^a^ Other 1L regimens combined is comprised of the following Line 1 regimens: CVP+R, R‐CHOP/R‐DHAP, and other (bendamustine, CHOP, BAC‐R, CHLO‐R, ibrutinib, rituximab).

### 3.5 Overall Survival

OS was influenced by line of therapy, therapeutic regimen, and patient age. The median OS (mOS) in 1L was 3.51 years (95% CI: 2.89, 4.03). This decreased with subsequent lines of therapy (0.60 years [95% CI: 0.40, 0.85] in 2L, 0.43 years [95% CI: 0.08, 0.62] in 3L) (Table 5).

**TABLE 5:** Median overall survival, by treatments received.

	Median (95% CI), years
1L treatment	3.51 (2.89, 4.03)
BR	4.02 (3.23, 4.72)
R‐CHOP	2.67 (1.52, 5.17)
Other^a^	1.04 (0.77, 3.28)
R‐CHOP/R‐DHAP	(S)
R‐CVP	1.06 (0.62, 3.96)
Other	2.02 (0.55, 3.58)
2L treatment	0.60 (0.40, 0.85)
Ibrutinib	0.50 (0.31, 0.85)
Other^b^	0.83 (0.42, 1.70)
Bortezomib‐based therapies	0.51 (0.09, 0.94)
Other	0.86 (0.42, 2.16)
3L treatment	0.43 (0.08, 0.62)

*Note*: (S) suppressed as a result of small cell.

Abbreviations: 1L = first line; 2L = second line; 3L = third line; BAC‐R = rituximab, bendamustine, and cytarabine; BR = bendamustine and rituximab; CHLO = chlorambucil; CHOP = cyclophosphamide, hydroxydaunorubicin, oncovin, and prednisone; R‐CHOP = rituximab, cyclophosphamide, hydroxydaunorubicin, oncovin, and prednisone; R‐CVP = rituximab, cyclophosphamide, vincristine sulfate, and prednisone; R‐DHAP = rituximab, dexamethasone, cytarabine, and cisplatin.

^a^ Other 1L regimens combined is comprised of the following Line 1 Regimens: CVP+R, R‐CHOP/R‐DHAP, and other (bendamustine, CHOP, BAC‐R, CHLO‐R, ibrutinib, rituximab).

^b^ Other 2L regimens combined is comprised of the following Line 2 Regimens: Bortezomib‐based therapies and other (R‐CVP, RCHOP, CHOP, Chlo‐R, BR) treatments

Among patients who received 1L therapy, the longest mOS from 1L treatment initiation was observed in those who received BR (mOS: 4.02 years, 95% CI: 3.23, 4.72), followed by R‐CHOP (mOS: 2.67 years, 95% CI: 1.52, 5.17; Figure 2a). Among patients receiving ibrutinib in 2L, the mOS was 0.50 years (95% CI: 0.31, 0.85) from 2L treatment initiation (Figure 2b).

**FIGURE 2:**
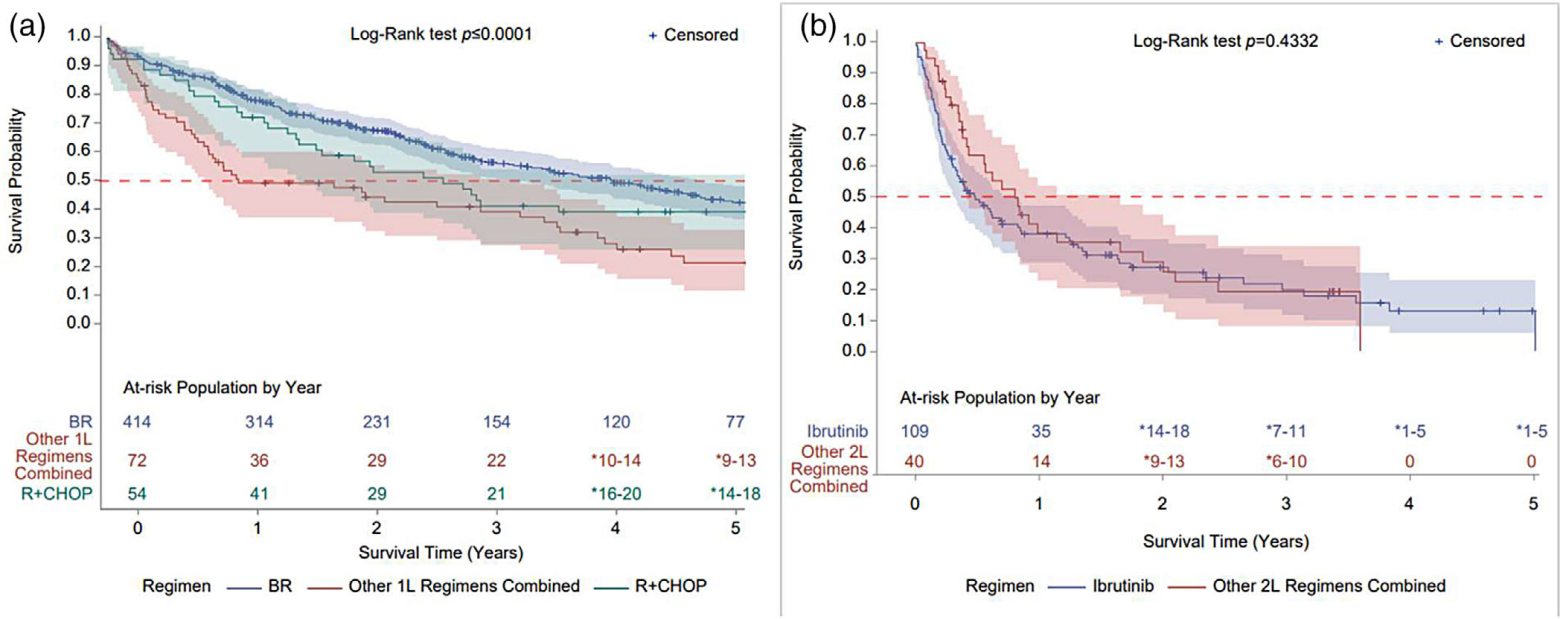
Overall survival from (a) 1L treatment initiation by 1L treatment regimen and (b) 2L treatment initiation by 2L treatment regimen. 1L = first line; 2L = second line; BR = bendamustine and rituximab; R‐CHOP = rituximab, cyclophosphamide, hydroxydaunorubicin, oncovin, and prednisone.

Among patients receiving 1L therapy, the mOS was lower in patients ≥ 75 years old (mOS: 2.49 years, 95% CI: 2.11, 2.82) than in patients 65–74 years old (mOS: 5.95 years, 95% CI: 4.26, 8.01) (Figure S4).

## 4 Discussion

Therapeutic options for treatment‐naïve and R/R MCL are limited, particularly for older patients who are ineligible for ASCT and CITs [18, 19, 20]. Combination chemotherapy and BTKis such as ibrutinib and acalabrutinib have shown promising efficacy as 1L therapy for older patients with MCL [4, 5, 15, 21, 22]. While previous studies have evaluated treatment patterns and clinical outcomes in MCL, focus has been on patients receiving therapy regardless of transplant eligibility [10, 23, 24, 25, 26]. Therefore, this publication sought to characterize the real‐world treatment patterns and outcomes in newly diagnosed patients ≥ 65 years old who were transplant‐ineligible in both 1L and R/R settings. Overall, this study found that the trend in crude incidence of transplant‐ineligible MCL remained consistent throughout the study period, which aligns with the reported stability of new cases of NHL in the United States between 2021 and 2022 [27, 28, 29, 30]. BR and R‐CHOP were the most commonly used treatments in 1L and ibrutinib was the most common in 2L. Mortality after 1L and 2L therapy was high, with 39% of patients dying after 1L therapy, as well most (68%) dying on 2L therapy. The duration of treatment, excluding rituximab maintenance therapy, was less than 5 months in 1L and just over 2 months in 2L. The mOS was low after 1L (3.5 years) and was further reduced in the R/R setting (7.2 months in 2L, 5.2 months in 3L). These findings suggest that new treatment options are needed to improve outcomes and prolong survival.

Given the clinical outcomes observed in the ECHO trial, the 1L treatment landscape in older patients with MCL is changing and new options are becoming available [16, 31]. At the time of writing, three BTKis (ibrutinib, acalabrutinib, and zanubrutinib) were approved for use in R/R MCL in Canada; however, only ibrutinib monotherapy is publicly funded [4, 32, 33, 34, 35]. In one Canadian study of patients ≥ 65 years old with newly diagnosed ASCT‐ineligible MCL between 2013 and 2016, the most commonly used 1L treatments were BR (78%) and R‐CHOP (13%) [10]. Our study had comparable findings (BR, 76%; R‐CHOP, 10%), highlighting the limited availability of novel treatments for elderly Canadian patients in the frontline setting [10]. These patterns also highlight the uptake of BR over R‐CHOP in patients with previously untreated MCL based on findings in the STiL trial [13]. Similar treatment patterns were reported in two other analyses [36, 37].

A retrospective study of patients with MCL and unknown transplant eligibility in the United States reported attrition rates increasing with line of therapy in patients ≥ 65 years old (1L: 24%; 2L: 28%; and 3L: 30%) [38]. Similarly, among patients with mixed transplant eligibility who received 1L treatment in a study of European databases, 29% of patients died without receiving 2L treatment [37]. However, comparisons with published evidence should be interpreted with caution given the paucity of data on mortality in an elderly population with transplant‐ineligible MCL. The current study reported a higher rate of mortality after 1L therapy (39%) and 2L therapy (69%), as may be expected for elderly patients who are transplant‐ineligible. Mortality rate estimates may have been influenced by patient censoring; in the current study, 391 of 754 (52%) patients received 1L treatment only and did not move on to 2L treatment, of which 212 (54%) patients died following 1L. Only 125 of 754 (17%) patients received both 1L and 2L treatment with no subsequent therapy, of which 102 (82%) died following 2L. When considering censoring due to study end, the true probabilities of death and of receiving subsequent lines over longer follow‐up could differ from observed proportions and true mortality could be higher than estimated. These findings highlight the high mortality and limited progression to subsequent therapies among older patients with transplant‐ineligible MCL.

Elderly patients with transplant‐ineligible MCL may be unable to prolong survival with available treatment options. One US‐based study of elderly patients with R/R MCL and mixed transplant eligibility reported low real‐world mOS after 1L therapy that decreased further with subsequent treatment (after 1L: 4.5 years; after 2L, 1.8 years; after 3L, 1.0 year; after fourth‐line, 0.65 years) [36]. Notably, longer mOS (25 months) was observed in a pooled analysis from open‐label studies of patients with MCL with a median age of 68 years receiving ibrutinib, suggesting external factors contributing to truncated real‐world survival [39]. Results from the current study underscore the low mOS among the cohort after 1L treatment initiation (mOS: 3.5 years), with only half of these patients receiving rituximab maintenance therapy. The mOS was even lower following 2L treatment (mOS: 0.59 years). In Canada, rituximab maintenance therapy is approved for funding in all provinces for patients with indolent B‐cell lymphomas who have responded to chemoimmunotherapy [40]. The low uptake of rituximab maintenance therapy in this cohort may be due to the limited robust evidence on its efficacy in elderly patients. Further, given that older patients often have comorbidities, they may not respond to therapy or discontinue due to toxicity, suggesting they may be less likely to receive maintenance therapy. Current treatments may be ineffective at adequately managing MCL, which can be further exacerbated when patients are also ineligible for transplant.

Strengths of this study include comprehensive, linked, population‐level data encompassing nearly all patients diagnosed with MCL in the OCR. However, few patients treated through compassionate programs may not be captured, though this subset is not expected to impact analysis. In addition, given the focus on a single province, findings may not be fully generalizable to other provinces with differences in treatment access and clinical practices. While practice patterns may vary, the funding criteria for MCL treatments are generally consistent across provinces. Furthermore, censoring at study end may have limited assessment of patients diagnosed toward the end of the study period. Finally, given that PFS could not be assessed, TTNT‐D was used as a pragmatic surrogate endpoint as progression events are not reliably captured in health administrative data.

## 5 Conclusions

This is the first study reporting real‐world treatment patterns and clinical outcomes in both 1L and R/R settings of elderly patients with MCL who are transplant‐ineligible in Ontario. The most common treatments were BR and R‐CHOP in 1L and ibrutinib in 2L. Most patients receiving 1L died prior to receiving 2L treatment, and of patients who received 1L and 2L, nearly three‐quarters died following 2L treatment. Real‐world survival was low in 1L and even lower with subsequent treatment. This study highlights an unmet need for treatments that effectively manage elderly patients with MCL who are transplant‐ineligible.

## Author Contributions

S.G. was responsible for writing, review, and editing, conceptualization, methodology, project administration, and validation.  S.J.S. was responsible for writing, review, and editing, methodology, project administration, and validation. S.T. was responsible for writing, review, and editing and conceptualization. A.W. was responsible for writing, review, and editing, conceptualization, and validation. M.A.H. and A.P. were responsible for writing, review, and editing, methodology, and conceptualization.

## Funding

This study was sponsored by AstraZeneca Canada.

## Ethics Statement

The study was approved by the Sunnybrook Health Sciences Centre Research Ethics Board (REB #6410).

## Consent

The authors have nothing to report.

## Conflicts of Interest

Sydney George and Mackenzie A. Hamilton are employees and shareholders of AstraZeneca. Sandra Thibault contributed to this work while employed at AstraZeneca. Anca Prica has received consultancy fees from AstraZeneca, Kite Gilead, and Roche. Soo Jin Seung and Anisia Wong have no conflicts of interest to declare specific to their work on TRACE.

## Supporting information



Supporting Information File 1

## Data Availability

The data that support the findings of this study are available from the Institute for Clinical Evaluative Sciences. Restrictions apply to the availability of these data, which were used under license for this study.
