# Supplementary material for: Real‐World Treatment Patterns, Burden of Illness, and Survival of Autologous Stem Cell Transplant‐Ineligible Patients With Mantle Cell Lymphoma in Ontario, Canada
Source: EJHaem. 2026 Oct 15;7(5):e70384. doi: 10.1002/jha2.70384 (PMC13670415; doi:10.1002/jha2.70384)
Supplement: Supplementary file 1 [file JHA2-7-e70384-s001.docx]

**Title:** Real-world treatment patterns, burden of illness, and survival of autologous stem cell transplant-ineligible patients with mantle cell lymphoma in Ontario, Canada

**Running title**: Real-world treatment patterns and clinical outcomes in MCL

**Authors:** George, S.^1^, Seung, S.J.^2^, Thibault, S.^3^, Wong, A.^2^, Hamilton, M.A.^1^, Prica, A.^4^

**Affiliations:**

1. Scientific Affairs, BioPharmaceuticals, AstraZeneca, Mississauga, Ontario, Canada
2. Health Outcomes and PharmacoEconomics (HOPE) Research Centre, Sunnybrook Research Institute, Toronto, Ontario Canada
3. Medical Affairs, Oncology, AstraZeneca, Mississauga, Ontario, Canada
4. University Health Network, Toronto, Ontario, Canada

**Abstract**

Since the approval of ibrutinib in 2016 in Canada for relapsed or refractory (R/R) mantle cell lymphoma (MCL), there has been limited evidence characterizing treatment patterns, burden of illness, and survival in Canadian patients ineligible for autologous stem cell transplant (ASCT). The aim of this study was to characterize the real-world use and outcomes of patients with MCL who were ≥65 years old and transplant-ineligible in first-line (1L) and R/R settings between 2013 and 2021 using administrative databases in Ontario, Canada. Incidence of MCL, treatment patterns, and overall survival (OS) were assessed. The study cohort consisted of 754 patients (follow-up: 36.1 months, median age: 76 [IQR: 71-82], 71.1% male). Most patients received bendamustine and rituximab in 1L (76.7%) and ibrutinib in second-line (2L; 73.2%). Mortality rates following 1L and 2L therapy were 39.3% and 68.5%, respectively. Duration of therapy in 1L and 2L treatment was 4.70 months and 2.14 months, respectively. Median OS decreased with subsequent lines of therapy (1L: 3.51 years; 2L: 0.60 years; third-line: 0.43 years). This is the first study in Ontario reporting real-world treatment patterns and clinical outcomes of elderly patients with MCL who are transplant-ineligible in 1L and R/R settings.

**Keywords:** mantle cell lymphoma, real-world evidence, transplant-ineligible, treatment patterns, clinical outcomes, burden of illness

# 1.0 Supplementary Material

**Supplementary Table 1: Description of Data Sources**

| **Database** | **Description** |
| --- | --- |
| Ontario RPDB | Provided basic demographic information (age, sex, location of residence, date of birth, and date of death for deceased individuals) for those issued an Ontario health insurance number. |
| OCR | Contained information on all Ontario residents who have been newly diagnosed with cancer ("incidence") or who have died of cancer ("mortality"). |
| ODB | Contained prescription medication claims for those covered under the provincial drug program, mainly: those aged 65 years and older, nursing home residents, patients receiving services under the Ontario Home Care program, those receiving social assistance, and residents eligible for specialized drug programs. |
| COVID-19 Integrated Testing Dataset | A comprehensive dataset of all available COVID-19 diagnostic laboratory results in Ontario. |
| CONTACT | Contained indicating markers for Ontario residents who were eligible, had any health care contact, and/or enrolled in Client Agency Program Enrolment (CAPE) during each quarter of a calendar year. |
| New Drug Funding Program | Captured the use of new, often expensive, cancer drugs. |
| Activity Level Reporting | Represented the basic set of data elements required to produce the quality, cost, and performance indicators for the cancer system. |
| OHIP | Contained physician services, diagnostic and laboratory procedures. |

Supplementary Figure 1: Study design

Abbreviations: ADG = aggregated disease group; CCI = Charlson Comorbidity Index; MCL = mantle cell lymphoma

Supplementary Figure 2: Patient flow diagram

Abbreviations: IKN = ICES Key Number; MCL = mantle cell lymphoma; OHIP = Ontario Health Insurance Plan.

Supplementary Table 2: Time from diagnosis to treatment and duration of treatment in patients ≥65 years old diagnosed with MCL between 2013 and 2021, by line of therapy

|  | **Median (IQR), months** |
| --- | --- |
| Time from diagnosis to 1L treatment initiation | 1.64 (0.99-3.88) |
|  |  |
| **Duration of 1L treatment** |  |
| Including rituximab maintenance | 12.8 (4.64-26.4) |
| Excluding rituximab maintenance | 4.70 (3.68-5.33) |
|  |  |
| Time from diagnosis to 2L treatment initiation | 21.6 (11.1-38.9) |
|  |  |
| **Duration of 2L treatment** | 2.14 (0.79-5.95) |

Abbreviations: 1L = first line; 2L = second line; IQR = interquartile range. MCL = mantle cell lymphoma.

Supplementary Figure 3: Time to next treatment or death from 1L treatment initiation, by 1L treatment regimen

Abbreviations: 1L = first line; R-CHOP = rituximab, cyclophosphamide, hydroxydaunorubicin, oncovin, and prednisone.

Supplementary Figure 4: Overall survival from 1L treatment initiation for all 1L regimens, stratified by age at 1L initiation

Abbreviations: 1L = first line.
